# Communication practices in the US and Syria

**DOI:** 10.1186/s40064-016-2486-9

**Published:** 2016-06-23

**Authors:** Rebecca S. Merkin, Reem Ramadan

**Affiliations:** Baruch College, CUNY, 1 Bernard Baruch Way, New York, NY 10010 USA; Damascus University, Damascus, Syria

**Keywords:** Cross-cultural differences, Intercultural relations, Individualism, Collectivism, Ingroup, Outgroup, Communication adaptability, Non-verbal immediacy, Social self-efficacy (SSE), General self-efficacy (GSE), Empathy, Culture, Syria

## Abstract

This study highlights Syrian communication practices using comparative tests with the United States communication as a baseline. Additionally, theoretical findings on individualism and collectivism theory are extended to include findings from Syria. Multivariate Analysis of Covariance was used to test culture’s effect in demographically similar (in age, SES, and education) student convenience samples, with the covariate communication adaptability, on dependent variables: empathy, social confirmation, social composure, friendships, non-verbal immediacy, social self-efficacy, and general self-efficacy. Results indicated that Syrians possess more empathy, social confirmation, and perceived general self-efficacy in comparison to U.S. citizens who have greater social composure, friendships, non-verbal immediacy and social self-efficacy. These results indicate that Syrians have the strength of self-efficacy to succeed in intercultural relationships while U.S. Americans have the assets of warmth and sociability to enable successful interactions with Syrians.

## Background

Presently, the world is in flux. Unrest, instability, and change are rampant in many nations. Internal political disruptions and external conflicts over Syria have made it the center of attention of some of the most powerful lobbies and countries in the world. What’s more, calls have been made to settle Syrian refugees in Europe and the United States (Chakraborty 2015; Tausch 2015). Therefore, this study attempts to advance the field of communication to include a better understanding of differences in communication between collectivistic Syria and individualistic cultures such as the United States in order to forge alliances in the more integrated societies of the future.

In fact, the future is approaching. According to the State Department, the United States has begun receiving and expects thousands more refugees from Syria in 2016 and beyond, despite concerns about foreign fighters (Jones 2015). Past and upcoming contact highlights the variance of perspectives between members of different cultures such as Syria and the United States. It is inevitable that differences in cultural perspectives will be channeled through communication. If communication is to be productive, interactions based on cultural knowledge and mutual respect will be needed to encourage Syrian and American relations to progress. Although there are numerous reasons for intercultural contact, misunderstandings between members of different cultures tend to occur less for political reasons than for cultural differences in values, norms, and negotiation styles (Chang 2003). Whatever the reason, a better understanding of differing cultural communication patterns could help intercultural interactions flow in multiple contexts.

In particular, cultural differences in empathy, social confirmation, social composure, friendships, non-verbal immediacy, social self-efficacy, and general self-efficacy will be addressed because of their centrality in the successful flow of communication. For example, empathy is generally understood to be a responsiveness to another’s experience. Empathy has beneficial effects on interpersonal communication (Batson et al. 2002) including an improved communication climate, greater shared meaning, and increased nonverbal connections (DiBacco 2008). Social confirmation is essential for human dignity and is a key to allowing others to uphold their *face* in the course of intercultural interactions (Honneth 2004; Zaharna 1991). Social composure or the maintenance of the others’ projected social image is directly related to relationship satisfaction (Lopez et al. 2007) and interpersonal maintenance behaviors, which are also essential to interpersonal relationship satisfaction (Weigel et al. 2016; Weiser and Weigel 2016). Those with more social self-efficacy tend to have better cognitive, affective, and behavioral communication skills as well as invest more effort and persist at relationships (Erozkan 2013; Schwarzer 2014). Social self-efficacy has also been demonstrated to lead to the greater use of positive (compromise, negotiation) as opposed to negative (attacking, power assertion) conflict resolution strategies (Field et al. 2014) as well as better interpersonal problem-solving skills (Erozkan 2013). Finally, general self-efficacy is the belief in one’s competence to tackle novel tasks and to cope with adversity in a broad range of stressful or challenging encounters (Luszczynska et al. 2005). General self-efficacy has been shown to be positively related to optimism, self-regulation, and self-esteem, and negatively related to depression and anxiety (Luszczynska et al. 2005).

The other variables explored and that are important to successful communication are friendliness and nonverbal immediacy. Cross-group friendships facilitate social interactions of immigrants with other members of a receiving society and are essential for the growth of constructive attitudes toward participation in the life of a receiving society (Ramelli et al. 2013). Nonverbal immediacy or warmth, encompasses behaviors that reflect the degree of psychological distance between (or closeness with) others, and includes behaviors such as head nods, eye contact and forward body lean (Andersen and Andersen 2005). Nonverbal immediacy provides emotional support and engagement (Jia 2015). Moreover, nonverbal immediacy provides supportive interactions that communicate ones’ intention to approach (as opposed to avoid) others (Jones and Wirtz 2007) and signals connection, attentiveness, and responsiveness (Coker and Burgoon 1987). Given the importance of the preceding variables to successful communication practices, the goal of the present study is to compare and highlight differences between communication dynamics in the United States and Syria. Highlighting differences in communication between the United States and Syria will aid in developing strategies to bridge differences between those from the United States and Syria. Understanding differences in individualistic and collectivistic values can also help individuals from both orientations to strategize when considering how to communicate with those from cultures other than their own.

## Individualism/collectivism

Individuals living in individualistic or collectivistic societies are expected to follow the norms associated with their respective cultures. Hofstede’s (2001) widely-known framework for studying culture includes four cultural dimensions [i.e., individualism/collectivism, uncertainty avoidance, power distance, and masculinity]. The individualism/collectivism dimension is considered to be the most powerful in explaining attitudes and practices than other cultural dimensions (Taras et al. 2010). For the purpose of comparison, the United States which is individualistic will be contrasted with Syria which is collectivistic (Hofstede 2001; Merkin and Ramadan 2010).

*Individualism*/*collectivism* describes the connection individuals have with their group. In individualist societies, “people prefer to act as individuals rather than as members of groups” (Hofstede 1994, p. 6). In collectivistic societies “people from birth onwards are integrated into strong, cohesive ingroups, which throughout people’s lifetime continue to protect them in exchange for unquestioning loyalty” (Hofstede 2001, p. 225). Groups’ goals such as family and business are a priority in collectivistic cultures such as Syria, while individual goals are emphasized more than group goals in individualistic cultures such as the United States. (Smith 2012). In fact, some languages (e.g., Arabic) do not employ the personal pronoun “I”, showing that collective identity is central. *Face* is of particular concern to those from collectivistic cultures as opposed to their individualistic counterparts (Merkin and Ramadan 2010). Consequently, research further indicates that collectivism is associated with high-context (implicit), indirect, communication while individualism is associated with low-context, direct communication (Gudykunst and Ting-Toomey 1988; Hall 1976; Park and Guan 2009). Although cultural level data indicates that most citizens of a particular culture possess similar values, it should be noted that within the same culture substantial individual variation is present. Accordingly, not all people from the same culture respond in the same ways (Park et al. 2012). Any analysis of individualism/collectivism should include theory relating to high/low context communication.

## Low context/high context communication, empathy, and social confirmation

Triandis’ (1988) theory of individualism/collectivism explains the values behind Hall’s (1976) theory of low-context and high-context cultures. More specifically, because ingroups are important to collectivists, they are more likely to engage in high-context communication (Smith 2012). Hall (1976) termed *low*-*context communication* as occurring when “the mass of information is vested in the explicit code” (Hall 1976, p. 70) and *high*-*context communication* as occurring when “most of the information is either in the physical context or internalized in the person, while very little is in the coded explicit transmitted part of the message” (Hall 1976, p. 79). While high- and low-context communication are used by all cultures, one practice tends to be predominant.

Members of individualistic cultures are inclined to use low-context communication (Gudykunst and Ting-Toomey 1988) and tend to communicate directly (Park and Guan 2009). For example, findings show that Americans are more likely to rate direct statements as effective in making a request (Kim and Bresnahan 1994). In contrast, members of collectivistic cultures usually use high-context communication while maintaining group harmony by communicating indirectly (Gudykunst and Ting-Toomey 1988; Kim and Park 2015; Merkin 2015).

For example, those from high-context cultures prefer less talk and are comfortable with silence (Allen et al. 2014). Collectivism has been shown to have a positive association with silence as well (Jaehoon et al. 2014). Collectivists also use more subtle situational cues, such as age and status when they communicate (Sadri 2014). Owe et al. (2013) have developed *contextualism*, a construct similar to high-context communication. Contextualism is the perceived importance of the context in understanding people; including social and relational contexts, such as family and social positions, but also physical environments (Owe et al. 2013).

Contextualism is highly correlated with interdependence, ingroup collectivism, and trust (Owe et al. 2013). Because of the implicit nature of contextual communication, members of high-context cultures have a need to establish social trust in personal and business relationships. To establish trust, negotiations in high-context cultures tend to be slow and ritualistic, and agreement tends to be based more on trust than on written documents (Sadri 2014).

Trust is partly developed in relationships through feelings of empathy. The more we empathize, the more we feel that the other person is like us, then trust follows (Levenson and Ruef 1992). Researchers found that Russian managers (collectivistic and high-context) had greater empathy than their U.S. American (individualistic and low-context) counterparts (Matveev and Nelson 2004). Given this previous finding and research supporting collectivists’ greater need for trust in relationships, the following hypothesis is posed:

### **Hypothesis 1**

Syrians will communicate using more empathy than U.S. Americans.

A similar construct related to empathy is social confirmation. Social Confirmation refers to the extent to which an individual exhibits verbal and non-verbal support for the self-image of another person. Social confirmation demonstrates a knowledge and understanding of differences and expresses support towards others (Duran 1992). Those from high-context cultures (e.g., Syrians) tend to read nonverbal communication better than those from low-context cultures (e.g., U.S. Americans) who require explicit cues (Hall 1976). Therefore, the following hypothesis is posed:

### **Hypothesis 2**

Syrians will communicate with greater social confirmation than U.S. Americans.

Individuals in high-context cultures tend to establish relationships at earlier ages and maintain them for life (Smith 2012). Consequently, those from high-context cultures are unaccustomed to ambiguity—particularly when associating with strangers. In general, reducing uncertainty is more important in high-context than in low-context cultures (Gudykunst 1983). A reflection of this sense of uncertainty with strangers or outgroups is the tendency for members of high-context cultures to make ingroup/outgroup distinctions (Triandis 1988). Specifically, in high-context societies people tend to cooperate with members of ingroups and compete with everyone else (Triandis 1988).

### Ingroup/outgroup distinctions, social composure, and friendship

Although individualistic and collectivistic cultures both distinguish between ingroups and outgroups, ingroups exert more influence in collectivistic relationships (Forbes et al. 2011). For example, in the collectivistic Arab world (Hofstede, 2001), people are clearly divided into friends and strangers (Nydell 2006). Generally, research indicates that collectivists tend to be less concentrated on specific friendships and more concentrated on their integration into their community and social networks (French et al. 2005).

Moreover, ingroups are more important to collectivists partly because their sense of selves tend to be more *interdependent* with their group, unlike their individualistic counterparts, who tend to see themselves as more *independent* entities apart from their group (Markus and Kitayama 1991). While independence and interdependence are constructs occurring on the individual level, the cultural-level equivalent to these constructs are Hofstede’s individualism and collectivism as well as Schwartz’s (2004) autonomy and embeddedness. Independence and embeddedness are both constructs that impact on the need for reducing uncertainty when communicating with outgroups (Schwartz 2004).

Besides collectivists’ tendency to express competiveness towards their outgroups (Triandis 1988), findings show that both verbal and physical aggression are more common with outgroups than ingroups among collectivists’ than individualists (Forbes et al. 2011). This is likely due, in part, to their uneasiness about dealing with unknown persons and contexts (Samochowiec and Florack 2010). On the other hand, individualists seem to be less likely to avoid uncertainty and are more trusting in relationships generally, because they tend to emphasize their own goals over those of their group (Smith 2012). The more relaxed posture of individualistic U.S. Americans makes it more likely that they have an open attitude toward social communication.

Duran’s (1992) social composure construct relates to the level of anxiousness an individual experiences in new social settings, thereby affecting the initial communication abilities one possesses. Specifically, *social composure* refers to the extent to which an individual feels relaxed and can manage anxiety positively (Duran 1992). While collectivists are likely to have more social confirmation characteristics which are other-oriented, individualists—who are more self-oriented—should be more likely to have greater social composure (Hales 2006).

Individualists should also be more likely to advance friendships because of their greater willingness to develop relationships with both ingroups and outgroups (Triandis 1988). Given their focus on self-promotion, U.S. individualists are also more likely to view themselves to be more capable of establishing relationships than those from high-context, collectivistic cultures such as Syria. An example of this is the case of United States youth, who when compared to youth in many other cultures, in their quest for autonomy, first and foremost put their faith in their friends (Schneider 1998). Consequently, the following hypotheses are posed:

#### **Hypothesis 3**

U.S. Americans will communicate with greater social composure than Syrians.

#### **Hypothesis 4**

U.S. Americans will develop more outgroup friendships than Syrians.

### Direct versus indirect communication, immediacy, and social self-efficacy

Collectivism has been associated with greater interpersonal solidarity experienced with ingroups (Triandis 1988), thus placing a stronger emphasis on indirect high-context communication (Allen et al. 2014). Collectivists tend to use indirect communication  out of concern for communication partners’ feelings, as well as concern with their own self-presentation to save face (Kim and Park 2015; Pavlidou 2008).

Since collectivists value group harmony (Kim and Park 2015), direct inquiries can be considered potentially face-threatening acts (Forbes et al. 2011). Face-threatening acts are of particular concern to collectivists who tend to be more sensitive to saving face and potential conflict. Consequently, high-context collectivists prefer less talk and are more comfortable with silence (Allen et al. 2014). Such indirect communication tends to maintain social harmony (Rojjanaprapayon et al. 2004). In fact, not stating clearly what one has in mind is considered to be a sign of strength, maturity, and social competence in high-context collectivistic cultures (Rojjanaprapayon et al. 2004). In contrast, in the individualistic United States, where the prioritization of the self over aspects of community is the norm, directly affirming one’s views is considered more powerful (Hales 2006).

The individualistic presentation of self is also characterized by direct nonverbal expressions of warmth or nonverbal immediacy. Mehrabian (1971) defined immediacy as “the degree of directness and intensity of interaction between a communicator and the object of his communication” (p. 414). Displays of immediacy behaviors are enacted through nonverbal communication including close proximity, gazing, smiling, and touching (Hinkle 1999). Nonverbal immediacy is also considered to be a pro-social communicative construct (McCroskey et al. 2006) that plays a significant role in U.S. daily communication interactions (Myers and Ferry 2001) and in relationship satisfaction (Sidelinger et al. 2012). Communicating immediacy promotes intimacy and psychological closeness as well as perceptions of receptivity/trust, similarity, and equality (Myers and Ferry 2001). Given U.S. American individualism, their low need to reduce uncertainty, their outgoing nature, and their direct communication patterns (Pavlidou 2008), it also likely that U.S. Americans are more verbally and nonverbally immediate. Consequently, the following hypothesis is posed:

#### **Hypothesis 5**

U.S. Americans will communicate with more nonverbal immediacy than Syrians.

Another factor which guides behavior during communication is having faith in one’s sociability or *social self*-*efficacy* (Fan and Mak 1998). Social self-efficacy refers to a willingness to initiate communication in social situations (Sherer and Adams 1983), including social tasks relating to making friends, social assertiveness, pursuing romantic relationships, performance in public situations, and receiving and giving help (Fan et al. 2012). Part of the reason individuals with strong perceived social self-efficacy are willing to approach others is that they also tend to have positive views about themselves (Fan et al. 2012). Additionally, those with high social self-efficacy tend to have more effective social behavior, psychological adjustment, and psychological health (Iskender and Akin 2010), psychological well-being (Liu et al. 2008), and a tendency to use more open communication (Maddux and Gosselin 2003).

Those from high-context cultures (e.g., Syria) also tend to experience more psychological distance in intercultural interactions than those from low-context cultures (Allen et al. 2014) such as U.S. Americans. As a result, U.S. individualists tend to feel less anxiety about new relationships and are more likely to feel confident about openly communicating with new people. Given that individualists believe they have control over their own destiny (Bandura 2001), they should also have greater confidence in their ability to engage in the social interactional tasks necessary to initiate and sustain relationships (Smith and Betz 2000) than their Syrian collectivistic counterparts. Thus, the following hypothesis is posed:

#### **Hypothesis 6**

U.S. Americans will have greater social self-efficacy than Syrians.

### General self-efficacy

Calls have been made to further examine the relationship between general self-efficacy and individualism/collectivism across nations because of dissimilar results (Roos et al. 2013). For example, past research has found the relationship between individualism/collectivism and general self-efficacy to not be significant (Wu 2009). On the other hand, studies comparing levels of self-efficacy and different cultural groups (e.g., Scholz et al. 2002) show that self-efficacy beliefs are characteristically higher for participants from Western, individualist cultures than they are in collectivist cultures (Klassen 2004). Although social factors are likely to be important to individualists, who are more outgoing, open, and less uncertain, more extreme uncertainty could cause collectivists to want greater control. Self-efficacy could also be related to individualism because it is more focused on the individual (Klassen 2004). However, because “a strong sense of efficacy is vital for successful functioning regardless of whether it is achieved individually or by group members working together” (Bandura 2001, p. 16), all cultures could be likely to possess self-efficacy. Accordingly, others believe that self-efficacy is not culturally determined.

Exerting control over events affecting one’s life provides agency over unsettling uncertainty. In fact, a recent study indicates that valuing a sense of collectivism increases the likelihood that individuals will engage in assertive behaviors in their organization (Love and Dustin 2014). According to Bandura (1997), efficacy beliefs, defined as “beliefs in one’s capabilities to execute the courses of action required to produce given attainments” (Bandura 1997, p. 3) provide people with a self-motivating mechanism that mobilizes efforts to target behavior in the direction of goals. To the extent that people are able to control their outcomes, they are also better able to predict them (Bandura 2000). Predictability fosters adaptive readiness. By influencing events over which people have control, people can better realize desired outcomes and forestall undesired ones. In contrast, the inability to exert influence over the things that adversely affects one’s life breeds apprehension, dysfunction, apathy, and despair (Bandura 2000). In fact, evidence shows that locus of control, neuroticism, and generalized self-efficacy are all related concepts (Judge et al. 2002). Given that results are presently inconclusive and competing rationales exist, the following question is posed:

#### Research Question 1

Will Syrians have greater general self-efficacy than Americans?

## Method

### Participants

The participants in this study were college students studying in their home countries in business schools within the same age range. The students in Syria were studying in Damascus and students in the United States were studying in New York City—both urban environments. The U.S. sample were composed of many first generation Americans who were born in the United States but have immigrant parents. The Syrian participants were all born in Syria and have Syrian parents. The U.S. student population reflected the ethnic composition of a city-school in New York City in that the majority of the sample were European Americans (*n* = 79), followed by Asian Americans (*n* = 34), Indian Americans (*n* = 16), African Americans (*n* = 10), Hispanic Americans (*n* = 10), Italian Americans (*n* = 8), Russian Americans (*n* = 6), and Middle-Eastern Americans (Turkish, Jordanian, Greek, and Persian). The mean age of the U.S. students was 23 (*SD* = 3.25) and the mean age of the Syrian sample was 21 (SD = 2.25). Syrian students were 55 % male and 45 % female while the U.S. students were 44 % male and 56 % female.

### Procedures

The questionnaire was developed in English and translated into Arabic as well as back-translated by different bilingual scholars to ensure linguistic equivalence (Brislin 1986). U.S. participants received the questionnaire in English and Syrian respondents received the questionnaire in Arabic. The U.S. participants were recruited from a variety of communication classes and the Syrian participants were recruited from business classes. Participants were asked to respond to Likert-type questions measuring their empathy, social confirmation, social composure, friendships, non-verbal immediacy, social self-efficacy, and general self-efficacy. The questionnaire required approximately 15–30 min to complete. All the participants responded to the questionnaire in their native language. The participation was anonymous.

### Measures/instruments

Individualism was operationalized by country as per Hofstede’s (2001) theory. In addition we previously empirically tested both countries’ levels of individualism and collectivism and found that Syria is more collectivistic than the United States which is individualistic (Merkin and Ramadan 2010). Since it was not necessary, this variable was not measured again for this study.

Communicative adaptability was measured using Duran’s (1992) Communication Adaptability Scale. The overall Communicative Adaptability Scale in this study consists of 15 items such as, “I feel nervous in social situations”, “I try to be warm when communicating with others and “I enjoy meeting new people”. Generally, communication adaptability measures the ability to perceive socio-interpersonal relationships and to adapt one’s interaction goals and behaviors accordingly. The items were constructed as five-point Likert-type statements. The overall scale (Combined α = .85; United States α = .81; Syria α = .77) contains six subscales: social composure, social confirmation, social experience, appropriate disclosure, articulation, and wit. The two subscales most relevant to successful communication between members of different cultures were used in this study. In particular, they were Social Composure (the degree of anxiousness people feel in new social situations), 5 items (Combined α = .76; United States α = .88; Syria α = .77) and Social Confirmation (the degree of knowledge and understanding of differences and expression of support towards others), 5 items (Combined α = .85; United States α = .84; Syria α = .75).

Non-verbal immediacy was measured using Richmond et al.’ s (2003) Nonverbal Immediacy Scale (NIS) to see differences in the tendency to communicate with closer emotional distance and greater use of non-verbal behavior. Response options used a Likert-type scale with a 5-point variation: 1 = Never and 5 = Always. The scale consisted of 26 items such as, “I look over or away from others when they touch me while we are talking”. Reliabilities were as follows: Combined α = .70; United States α = .69; Syria α = .70.

Social self-efficacy was measured using the original Self-Efficacy Scale (Sherer and Adams 1983; Sherer et al. 1982) which consisted of two subscales: General Self-Efficacy (GSE) and Social Self-Efficacy. Only the Social Self-Efficacy subscale was used in this study which consisted of a six-item, 5-point Likert-type scale ranging from 1 = Strongly disagree to 5 = Strongly agree, since this was to tested more adequately by the GSE scale below (Combined α = .63; United States α = .70; Syria α = .60).

The ten-item GSE Scale (Schwarzer and Jerusalem 1995) modified by Scholz et al. (2002) to a nine-item scale was used to test general self-efficacy for this study. Answers were adapted to a Likert-type scale with a 5-point variation, ranging from 1 = Strongly disagree and 5 = Strongly agree instead of a range from 1 = Not at all True to 4 = Exactly True as indicated in the original scale. The alphas for the 9 items were as follows: Combined α = .83; United States α = .80; Syria α = .80.

Empathy was measured via a subscale from the Interpersonal Reactivity Index (IRI). According to Davis (1996), Empathic Concern (EC) measures other-oriented feelings of sympathy and concern for unfortunate others. Students answered EC items on a 5-point Likert-type scale ranging from 1 = Does not describe me well to 5 = Describes me very well. The scale consisted of items such as, “I often have tender, concerned feelings for people less fortunate than me”. Empathic Concern consists of seven items (Combined α = .63; United States α = .66; Syria α = .66).

Friendship communication was measured using an adapted scale from Hawthorne (2006). Four of the six items from the Friendship Scale, which was designed to assess social isolation, were used for assessing friendship behavior. Answers were modified to a Likert-type format with a 5-point variation ranging from 1 = Never to 5 = Always. The scale consisted of items such as, “It has been easy to relate to others” and the alphas were (Combined α = .66; United States α = .71; Syria α = .64).

### Statistical analysis

Differences in United States and Syrian communication were tested by means of a MANCOVA design with country as the independent variable, communication adaptability (which was a significant competing predictor) as the covariate, and non-verbal immediacy, empathy, social competence, social confidence, social self-efficacy, and general self-efficacy as the dependent variables. The sample size necessary for adequate power in the hypotheses using multivariate analyses is between 58 subjects per group (Lauter 1978) and 70 per group (Cohen 1988). Both samples had more than 70 participants.

## Results

Overall results showed that multivariate analysis was warranted because the multivariate main effect for culture was significant [Wilks’ α = .76; *F*(7,338) = 15.41, *p* < .0001, partial η^2^ = .24]. The covariate was significant [*F*(7,338) = 189.35, *p* < .0001, partial η^2^ = .80]. Univariate results and accompanying descriptive statistics are summarized in Table 1. The inter-correlations among the dependent variables can be found in Table 2.

**Table 1:** Analysis of variance summary, means, and standard deviations

Communication	US/Syria			US		Syria	
	*F*	*η* ^2^	*p*	*M*	*SD*	*M*	*SD*
Empathy	15.55	.040	.0001	3.63	.78	4.00	.68
Social confirmation	8.54	.020	.0040	3.76	.74	4.01	.68
Social composure	15.76	.040	.0001	3.85	.69	3.69	.78
Friendship	5.40	.020	.0200	3.89	.69	3.71	.90
Nonverbal immediacy	37.05	.100	.0001	3.63	.43	3.38	.38
Social self-efficacy	37.05	.100	.0001	4.18	.79	3.63	.98
General self-efficacy	37.51	.100	.0001	3.32	.42	3.74	.58

The overall effect and the covariate effects are in the results section

*M* mean, *SD* standard deviation

**Table 2:** Correlations among dependent variables

	Nonverbal immediacy	Social composure	Social confirmation	Social self-efficacy	General self-efficacy	Empathy	Friendship	Communication adaptability
Nonverbal immediacy	1	−.313**	.247**	.386**	.040	.145**	.398**	.358**
Social composure		1	−.198**	−.374**	−.260**	−.053	−.315**	−.645**
Social confirmation			1	.211**	.197**	.374**	.211**	.754**
Social self−efficacy				1	.212**	.120*	.413**	.373**
General self-efficacy					1	.061	.231**	.314**
Empathy						1	.109*	.304**
Friendship							1	.352**
Communication adaptability								1

** Correlation is significant at the .01 level (2-tailed)

* Correlation is significant at the .05 level (2-tailed)

Univariate results indicated that Syrians communicate using more empathy than U.S. Americans in support of Hypothesis 1. Support was also found for Hypothesis 2 in that Syrians communicated with greater social confirmation than U.S. Americans. Findings showed that U.S. Americans communicate with greater social composure than Syrians supporting Hypothesis 3 and that U.S. Americans will also communicate with more friendship goals than Syrians supporting Hypothesis 4. Hypothesis 5, that U.S. Americans will communicate using more nonverbal immediacy than Syrians was also supported. Finally, U.S. Americans had greater social self-efficacy than Syrians, indicating support for Hypothesis 6. Answering Research Question 1, results showed that Syrians had greater general self-efficacy than U.S. Americans.

## Discussion

The goal of the present study was to compare important communication factors necessary for successful interaction (i.e., empathy, social confirmation, social composure, friendships, non-verbal immediacy, social self-efficacy, and general self-efficacy) between people from the United States and Syria. The results of this study can help direct future communication between those from collectivistic Syria and the individualistic United States.

While this study looked at communication differences from the frame of individualism and collectivism, other cultural dimensions exist and these results do not preclude other cultural indicators. This study basically explored how collectivistic values such as using high-context communication indirectly allows for a greater probability of those communicating to save face (Constantine and Sue 2006; Merkin and Ramadan 2010). Additionally, this study examined communication likely to be used by individualists who tend to prefer direct communication because they are less concerned with losing face which they believe can be negotiated through interaction.

In keeping with indirect collectivistic communication with the aim of allowing all to save face, the results of this study showed how Syrians are more likely to communicate showing empathy towards the other and affirming others through social confirmation. In contrast, those from the individualistic United States were shown to communicate in a more outgoing manner, as reputed (Stephan et al. 1993) with friendship, expressed with nonverbal immediacy and social composure. This profile of a more outgoing person is consistent with the results showing that those from the United States have higher levels of social self-efficacy. Given the results showing that collectivistic Syrians have higher levels of general self-efficacy, they should be able to adapt to unfamiliar communication patterns given general self-efficacy’s link to optimism, self-regulation, and self-esteem (Luszczynska et al. 2005).

To Americans, being outgoing, friendly, and informal is considered good (Kohls 2011). However, those whose orientation is to save face might be concerned that they will be caught off guard by too much familiarity. Thus, it is important to note that successful communication from the United States side could require greater empathy, patience, and face-enhancing warmth than normatively is demonstrated during communication in U.S. culture. Therefore, it is particularly important to better understand these differences in communication before setting out for encounters with Syrian counterparts, whether they are government representatives, rebels, or refugees in the present and future.

Our findings also confirmed that U.S. Americans and Syrians have different communication assumptions and norms at play when they communicate. For example, during intercultural communication situations, inferences about a speakers’ intent are affected by the participants’ culturally specific use of contextualization cues and background knowledge (Kagawa-Singer and Kassim-Lakha 2003). Like collectivists, Syrians are likely to expect to share contexts (experiences) with people before considering them as their friends or before solidifying agreements. Therefore, such knowledge needs to be applied during communications between those from the United States and Syria. Accordingly, it would be worthwhile to consider actively engaging in activities and sharing social time together during interactions between U.S. Americans and Syrians before diving into the goal at hand.

### Implications and future research

As collectivists, Syrians are likely to communicate differently with their ingroups than their outgroups (Forbes et al. 2011). It would therefore, be useful to note that competitive or cold behavior might be an initial response to communication but this reaction could change and thaw over time after getting to know people. What’s more, it might be strategic for individualists to try to figure out how to become part of the in group of those they want to work with from Syria so that they can develop greater trust and warmth. The individualistic low-context assumption that words are most important (Hall 1976) is not likely to be shared by Syrians (Constantine and Sue 2006). Oftentimes, individualists could perceive collectivists as too silent, too tentative, and too vague, thus lacking authority (Kagawa-Singer and Kassim-Lakha 2003). However, attitudes that follow such assumptions could sabotage the relationship from continuing. Consequently, U.S. Americans need to keep an open mind when engaging in conversations with Syrians because negative attitudes could derail relational goodwill. Instead using their greater nonverbal immediacy and social self-efficacy to show interest in what’s stimulating about their cultural counterparts would be more fruitful. Future observational research to investigate the range of reactions of different cultural members as to what is considered appropriate versus what is considered inappropriate communication would be helpful to see the actual success of strategies attempted.

Other results of this study related to collectivistic high-context communication theory show that Syrians, like others from high-context cultures, tend to be more empathic and tend to express greater social confirmation in their relationships with others than U.S. Americans (Gudykunst and Nishida 1986). Empathy is an important element of communication competence (Matveev and Nelson 2004). In addition, social confirmation provides non-verbal support for the self-image of others making them feel important (Duran 1992). Researchers involved with intercultural training point out that “people can be encouraged to modify specific behaviors so that they are appropriate to the culture in which they find themselves and so that they will have a greater chance of achieving their goals” (Bhawuk and Brislin 1992, p. 414). However, knowledge of the types of miscommunication that could occur is important to know in order to increase conversers’ cultural sensitivity. Thus, while low-context U.S. Americans tend to focus on the importance of what is written and said, they also tend to be less attentive to what is *not* said. To be more competent at communicating with collectivists, it would be helpful for U.S. Americans to pay close attention to high-context communication factors as well as to emphasize their own nonverbal messages during their interactions with Syrians. While this assumption is deduced, future research is necessary to test the ways collectivists behave more other-oriented than individualists who tend to be more self-oriented.

Research shows that U.S. Americans are stereotypically perceived as optimistic, independent, outgoing, competitive, aggressive, emotional, friendly, and flexible (Stephan et al. 1993). Consistent with these perceptions and past theory, the findings of this study indicate that US Americans (on the cultural level) tend to be outgoing and tend to communicate with greater social composure, have more of an orientation towards befriending different people, and express greater nonverbal immediacy than Syrians. Their individualistic focus leads Americans to feel relaxed, composed, and comfortable when socializing with new people. For the most part, U.S. Americans find diverse individuals to be quite interesting and refreshing. For example, a majority of foreign students viewed U.S. Americans as being open when meeting them and as making an effort to get to know them (Rajapaksa and Dundes 2002). Then again, to some, those from the United States may still appear to be a bit overconfident and focused on themselves during intercultural interactions which individualism mandates. Consequently, U.S. Americans may not take into account that when there are fewer common values in a shared encounter, it is more likely to result in miscommunication (Kagawa-Singer and Kassim-Lakha 2003). Therefore, U.S. Americans can focus on training themselves to attend to others and engage in more active listening during intercultural interactions to accomplish their goals more successfully. Future studies testing the actual effects of active listening on goal attainment between individualists and collectivists is warranted.

Undeniably, the assumptions made on the basis of the significant results of this study are generalized expectations based on probabilities. There are also, of course, individual differences within cultures. However, one of the best ways of knowing how to plan for intercultural encounters is to look at differences, because differences are the most vulnerable areas likely to generate miscommunication (Moran et al. 2014). Nevertheless, acknowledging the other whether directly or subtly with goodwill can go a long way in bridging differences in the process of accomplishing mutual goals. Part of the knowledge gained in this study shows the typical blunders that are likely to occur automatically if individuals inadvertently enter into conversations with others from unfamiliar cultures. Knowing how to communicate and not offend can be accomplished keeping differences in mind and managing the appropriateness of one’s communication (as described above) during intercultural interactions. Future research is needed to test and rank order different communication strategies employed successfully between cultures.

Another one of the unique findings of this study is that Syrians have greater general self-efficacy than U.S. Americans while U.S. Americans have greater social self-efficacy than Syrians. Although this study attempted to theoretically advance findings emanating from individualism and collectivism in a cultural general fashion, self-efficacy appears to be more culturally specific. Indeed, calls have been made to further examine self-efficacy and individualism versus collectivism (Roos et al. 2013) because of dissimilar results across nations. Thus, in this particular study, social communicative factors appear to indicate that U.S. Americans who also have greater social composure, orientations towards friendships, and non-verbal immediacy are more willing to initiate communication in social situations (Sherer and Adams 1983).

While generally speaking self-efficacy beliefs are typically higher for participants from Western, individualist cultures as opposed to collectivist cultures (Klassen 2004), more extreme uncertainty such as intercultural interaction or outside environmental factors could be cause for collectivists to value the importance of greater control. Thus, the results of this study indicate that collectivistic Syrians possess a strong sense of general self-efficacy vital for survival and successful functioning which is perhaps achieved via group members working together as collectivism would mandate (Bandura 2001). Research is necessary to clarify the motives behind self-efficacy in order to better understand previously unexplained differences between those from individualistic versus collectivistic cultures.

### Limitations

Some researchers claim that individualism and collectivism has been defined in such general terms in the literature that it is missing a clear meaning (Oyserman et al. 2002)—largely creating uneasiness by some in the scholarly community who call to eliminate these labels in support of more accurate terms (Cohen 2009). This study addressed this concern by studying cultures whose individualism and collectivism were already verified in previous research (e.g., Hofstede 2001; Merkin and Ramadan 2010).

The samples used for this study were highly educated and very urban groups. Consequently, this study’s finding are somewhat limited in that it applies more specifically to urban demographics in practice. This is true particularly in Syria, where rural–urban differences are so great. Hence, care must be given to apply the results of this study. Additionally, future research is needed to test other populations in both the United States and Syria to confirm these findings.

Although the use of college students has been debated (Karahanna et al. 2002). College students can be an ideal population in which to study culture and communication for several reasons. First, students generally come from the same socio-economic levels, thereby controlling for possible competing predictors with culture. Thus, for matching samples, though college students are not representative of the entire population, they act as good representatives of culture. In the case of the Syrian sample, it could be possible that the situation of attending college, despite the outside uncertain atmosphere, could possibly have skewed their levels of self-efficacy. On the other hand, other studies have found high general self-efficacy in those from collectivistic cultures (Love and Dustin 2014). Alternatively, in the case of Syria, particularly because of the many refugees and dire circumstances of many in the population, it is an opportunity to even be able to reach residents of Syria, albeit students, to find out more about their communication preferences.

Two of the measurement scales used for this study (empathic concern and friendship) had particularly low reliabilities in the Syrian sample and one of the scales (empathic concern) had low reliabilities for both the Syrian and U.S. samples. This was perhaps due to greater variability of either the cross-culture instrument in that the scales were originally created for a Western population or perhaps this was due to the scale being used for a different culture whose meanings differ somewhat from Western meanings. Though the back-translation was adequate it is possible that the translation introduced more variability into this study’s findings. Moreover, there were a smaller number of questionnaire items than usual in both the empathic concern and friendship scales which could contribute to their lower reliability. In short, the Syrian version of the empathic concern and friendship scales were slightly lower than the norm of .70. The U.S. sample also had a lower than usual reliability on empathic concern which could indicate that more items needed to be added to the scale to increase its reliability. Thus, caution should be taken in interpreting results using these scales.

Finally, this study used the self-report method which could have limitations of possible social desirability effects. However, asking people what they are thinking is, in some cases, the only way to obtain needed information.

## Conclusion

This study showed that Syrians, exemplify a collectivistic high-context other-oriented culture that communicates with greater empathy, social confirmation, and perceived general self-efficacy than U.S. Americans. On the other hand, results showed that U.S. Americans communicate in line with their low-context individualistic cultural values. Specifically, communication patterns of U.S. Americans included greater social composure, friendship orientation, non-verbal immediacy, and social self-efficacy. During intercultural encounters it is important for different cultural members to achieve the cultural humility and goodwill required to respectfully take these communication differences into account in order to understand and work with others’ worldviews effectively in achieving our goals together with those from unfamiliar cultures (Alexander et al. 2014).
